# Real-time, spectral analysis of the arterial pressure waveform using a wirelessly-connected, tablet computer: a pilot study

**DOI:** 10.1007/s10877-018-0145-0

**Published:** 2018-04-28

**Authors:** David Andrew Pybus

**Affiliations:** St. George Private Hospital, 1 South St., Kogarah, NSW 2217 Australia

**Keywords:** Pulse wave analysis, Arterial pressure, Analysis, Fourier, Ventricular function

## Abstract

Spectral analysis of the arterial pressure waveform, using specialized hardware, has been used for the retrospective calculation of the ‘Spectral Peak Ratio’ (SPeR) of the respiratory and cardiac arterial spectral peaks. The metric can quantify the cardiovascular response to volume loading by analysing the effect of changing tidal volume (indexed to body weight) (V_TI_) on pulse pressure variability. In this pilot study, the feasibility of real-time SPeR calculation, using a mobile computer which was wirelessly connected to the patient monitor, was evaluated by examining the determinants of SPeR in 60 cardiac-surgical patients. In 30 patients undergoing aortic valve replacement (AVR), graded cyclical changes in ventricular loading were induced by increasing V_TI_ over 2 min, while performing spectral analysis at 1 Hz, before and after AVR. A strong, linear correlation between SPeR and V_TI_ was found and the slope of the regression line (β) changed significantly after AVR. The change in β correlated with the width of the preoperative vena contracta. In another 30 patients, SPeR at constant V_TI_ was calculated at 1 Hz during passive leg raising. β fell significantly on leg raising. The mean arterial pressure change during the manoeuvre was linearly related to the change in β. Real-time spectral analysis of the arterial waveform was easily accomplished. The regression of SPeR on V_TI_ was linear. β appeared to represent the slope of the cardiac response curve at the venous return curve equilibrium point. Measurements were possible at a significantly lower V_TI_ than the equivalent time domain metrics.

**Electronic supplementary material:**

The online version of this article (10.1007/s10877-018-0145-0) contains supplementary material, which is available to authorized users.

## Introduction

In patients undergoing intermittent positive pressure ventilation (IPPV), the assessment of pulse pressure variability (PPV) of the arterial waveform is useful for predicting the haemodynamic response to fluid loading [1–3]. It is usually analysed in the time-domain and is calculated in real-time by some patient monitors [4]. It is also well-accepted that PPV is significantly influenced by tidal volume and that there is a positive correlation between the tidal volume and the degree of PPV [5–8]. However, the precise nature of this relationship remains undefined.

Spectral (frequency-domain) analysis of PPV, using the fast Fourier transformation (FFT), has been reported occasionally [9, 10]. The technique is generally implemented using specialised hardware, but its real-time use in this clinical context has not been described. Unlike time-domain analysis, which can be performed on the heart beats found within a single respiratory cycle, in spectral analysis, it is necessary to analyse a sequence of breaths (typically 5–10) to quantify the low-frequency (ventilatory) data.

**Fig. 1:**
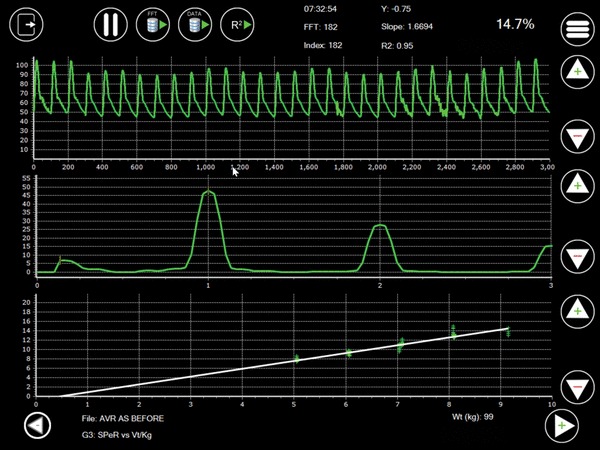
The application’s user interface. The upper display shows the current sample range of the arterial pressure waveform [Y = pressure (mmHg); X = sample number)]. The central display shows the most recently performed spectral analysis [Y = amplitude density; X = frequency (Hz)]. The respiratory and cardiac peaks and the second and third cardiac harmonics are clearly seen. The respiratory peak corresponds to a ventilator rate of 10 bpm and the cardiac peak to a pulse rate of 60 bpm. The lower display demonstrates the relationship between SPeR (%) and V_TI_ (ml kg^−1^). In this spectral display the current SPeR is 14.7%. The regression statistics have been calculated over the preceding 182 s and are shown at the top of the display. In this instance, the regression line has not been constrained to pass through the origin and the patient has been ventilated at five levels of V_TI_

The analysis effectively averages the frequency data contained within the sequence of breaths and provides a metric—the ‘Spectral Peak Ratio’ (SPeR) [9]—which can be used in the assessment of PPV. This ratio is calculated as the quotient of the respiratory and cardiac peaks—where the cardiac peak represents the total pressure development by the ventricle at end-expiration during the sample period and the respiratory peak represents the additional pressure development attributable to the change in stroke volume induced by ventilation (Fig. 1).

This pilot study was designed with two principal aims: to establish the feasibility of performing real-time spectral analyses of the arterial pressure waveform using a mobile computer; and to assess the clinical utility of such a technique.

Feasibility was assessed by modifying an existing ‘cross-platform’ application to which a real-time, spectral analytical module had been added [11].

The clinical utility of the application was then tested by examining its performance in 60 patients undergoing cardiac surgery. The utility study was designed to examine two hypotheses:

1. That the relationship between SPeR and tidal volume (indexed to body weight) (V_TI_) could be accurately described by a linear model of the form: SPeR = β * V_TI_
2. That the slope of this relationship (β) could be used to represent the slope of the cardiac response curve at its equilibrium point with the venous return curve in the ‘classical’ Guyton model of the circulation [12].

The first aim of the utility study was addressed by examining the relationship between SPeR and V_TI_, at various levels of tidal volume. In 30 patients, before and after aortic valve replacement (AVR), SPeR was measured during a respiratory manoeuvre where tidal volume was increased progressively between 5 and 9 ml kg^−1^. On completion of this manoeuvre, the regression of SPeR on V_TI_ and β were both calculated. The possibility of eliminating the need to perform a respiratory manoeuvre was also assessed by comparing this value of β with the value of β calculated simply as the quotient of SPeR and V_TI_ at a tidal volume of about 7 ml kg^−1^. The coefficient of determination (R^2^) was used to assess the goodness of fit with the simple linear model.

The second aim of the utility study was addressed by examining the effect of passive leg raising (PLR) on β, calculated at a constant tidal volume and high frequency. In 30 patients undergoing a variety of cardiac procedures, the effect of PLR performed over a period of about 150 s, during unchanging tidal ventilation, was studied. During PLR, spectral analysis was performed at 1 Hz. β was calculated on the completion of each analysis and recorded together with SPeR, V_TI_, mean arterial pressure (MAP) and central venous pressure (CVP). The relationship between the instantaneous changes in β and MAP which accompanied leg raising and lowering were then analysed.

## Methods

### Clinical study

60 paralysed, mechanically-ventilated patients undergoing cardiac surgery were studied. The institutional ethics committee approved the study (NSPHEC 2015-LNR-021) and informed consent was obtained from all participants. Two subgroups of patients were examined:

#### Subgroup 1

In 30 patients undergoing AVR, spectral analysis was performed at 1 Hz while graded, cyclical, changes in ventricular loading were induced by progressively increasing V_TI_ in a standardised manner (‘respiratory manoeuvre’). Testing was performed before sternotomy and after valve replacement and sternal closure. In this group, the pre-replacement vena contracta width (VCW) of the aortic regurgitant jet, measured in the oesophageal view, was also recorded. It was chosen for the assessment of the impact of preoperative aortic regurgitation on postoperative ventricular performance because it can be easily measured intraoperatively, is amenable to numeric analysis, and is a reliable indicator of the severity of aortic regurgitation [13].

#### Subgroup 2

In 30 patients undergoing a wide variety of cardiac surgical procedures, the effect of PLR on the SPeR, measured at 1 Hz and constant V_TI_, was measured. In this group, MAP and CVP were also recorded.

Patients were anaesthetised using a ‘balanced’ technique which included: Intubation and ventilation; the use of fentanyl and sevoflurane for the maintenance of anaesthesia and the use of pancuronium for muscle paralysis. Volume-controlled ventilation (VCV) was initiated using a tidal volume of 6–7 ml kg^−1^ at a frequency of 10–12 bpm. Monitoring was performed using an ‘Aisys CS2’ anaesthesia delivery system (GE Healthcare Inc., Madison, WI) and an ‘IE33’ ultrasound system (Philips Medical, Andover, MA).

### Signal acquisition and processing

The blood pressure signal was acquired from the radial artery using a 20 Ga cannula connected to a transducer by 1 m of manometer tubing. The transducer was fixed to the operating table at a height corresponding to the mid-axillary line and zeroed to atmospheric pressure before use. The signal was pre-processed using the monitor’s low-pass (22 Hz) filter and the digitised waveform was then continuously streamed from the monitor to the receiving computer at a sampling rate of 100 Hz.

### Computational techniques

A ‘cross-platform’ application, which acquired data wirelessly from the anaesthesia monitor in a manner previously reported [11], was created. The application accumulated waveform values in a ‘First in First Out’ (FIFO) buffer which was used as a ‘sliding temporal window’ on the waveform data. Once 3000 values (30 s range) had been acquired, the frequency spectrum of this temporal range was computed, and a cursor advanced along the FIFO buffer by 100 values. In this manner, each value contributed to up to 30 frequency spectra. During measurement sequences, analyses were performed repeatedly at 1 Hz. The application also acquired ‘basic’ physiological data, including the percentage systolic pressure variation, at 0.2 Hz.

In the spectral computation, three signal processing techniques were applied: the DC bias of the pressure waveform was minimized by subtraction of the mean value of the sample range from each value within the range before the transformation, a ‘Flat-Top’ window function [14, 15] was used for all transformations, and a high-pass filter (0.1 Hz) was applied to the transformed data.

The frequencies corresponding to the respiratory and cardiac peaks were then identified algorithmically and the SPeR was calculated as the ratio of the respiratory to the cardiac peak expressed as a percentage (Fig. 1). Inspired tidal volume, indexed to body weight (ml kg^−1^), was measured at the end of the sample range and also recorded. The sample range of 30 s permitted detection of a change in heart or respiratory rate of about two beats/breaths per minute.

Before clinical use, the analytical algorithms were refined using data synthesized by a purpose-written signal generator. The application was tested on various mobile computers running under the ‘Microsoft Windows 10’ ® and ‘Android 6’ ® operating systems. The application’s user-interface is shown in Fig. 1 and Video 1.

A complete specification of the signal acquisition system and computational environment, together with a ‘Microsoft Windows 10’ ® version of the application, can be found in the supplementary digital content.

### Respiratory manoeuvre

In the AVR subgroup, the respiratory manoeuvre was performed during stable anaesthesia, with the sternum closed, before and after valve replacement. VCV at a constant inspiratory flow rate and I:E ratio of 1:2 was used throughout the manoeuvre. The manoeuvre was initiated by reducing V_TI_ to about 5 ml kg^−1^ while performing continuous spectral analysis of the arterial pressure waveform and inspired V_TI_ at 1 Hz. After 45 s, V_TI_ was increased to about 7 ml kg^−1^ for 45 s and then to about 9 ml kg^−1^ for a further 45 s. Spectral analysis and data recording were then ceased and V_TI_ was restored to its starting value. The ventilation rate was not altered during the manoeuvre (Video 1).

### PLR

In the PLR subgroup, PLR was performed after induction of anaesthesia but before draping. The manoeuvre was initiated while performing continuous spectral analysis of the arterial pressure waveform at 1 Hz. Spectral analysis and data recording were commenced in the supine position. After about 45 s the patient’s legs were raised to about 45 degrees for about 45–60 s. They were then lowered back onto the operating table for a further 45 s. Spectral analysis and data recording were then ceased. Neither the ventilation rate nor V_TI_ were altered during the manoeuvre.

### Statistical analysis

Analyses were performed using ‘GraphPad Prism’ version 7.04 for Windows (GraphPad Software, La Jolla California USA). The complete ‘Prism’ datasets can be found in the supplementary digital content.

In the AVR subgroup, the SPeR: V_TI_ relationship was evaluated using linear regression modelling. The mean values of V_TI_ (ml kg^−1^) and SPeR (%) for each of the final 10 spectral analyses obtained at each of the three levels of V_TI_ were analysed. The analyses were constrained to model a linear regression through the origin. This technique was chosen for two reasons:

First, on physiological grounds, it is apparent that during apnoea (V_TI_ = 0 ml kg^−1^), no respiratory peak can be generated in the frequency spectrum and that the ‘correct’ value of the SPeR at a V_TI_ of 0 ml kg^−1^, must therefore be zero.

Second, if linear regression through the origin could be shown to accurately model the data, then the need to perform a respiratory manoeuvre in order to estimate the slope could be dispensed with (in the manner used in the PLR sub-group) and β could then be calculated at the time of each spectral analysis.

The slope of the SPeR: V_TI_ regression line (β) was calculated and the coefficient of determination (R^2^) was used to assess the ‘goodness of fit’ of the regression line. The significance of any difference in β between the ‘before’ and ‘after’ regression lines in individual cases was analysed using the extra-sum-of-squares F test. The 95% confidence intervals of the regression line were also calculated.

The significance of any difference between the grouped ‘before’ and ‘after’ values of β was assessed using the Wilcoxon matched-pairs signed rank test.

The measurement resolution of the technique at low tidal volumes was assessed by examining the change in SPeR which was found when V_TI_ was increased from the low- (~ 5 ml kg^−1^) to the mid-range (~ 7 ml kg^−1^) value in each of the 60 respiratory manoeuvres performed.

The significance of any difference between the values of SPeR obtained at low- and mid-range values of V_TI_ was assessed using the Wilcoxon matched-pairs signed rank test.

The possibility of eliminating the requirement to perform a ‘respiratory manoeuvre’ in order to calculate β was assessed in the 60 (‘Before’ and ‘After’) datasets by comparing the value of β derived from the respiratory manoeuvre with that calculated as the quotient of the mean value of SPeR found at the mean, mid-range value of tidal volume (~ 7 ml kg^−1^) in each case. This quotient corresponded to the slope of a line drawn between the SPeR at the mid-range tidal volume and the origin of the SPeR: V_TI_ graph i.e.:

$$\upbeta ={\text{SPeR}}/{{\text{V}}_{{\text{TI}}}}$$

In this manner, β could be calculated without the need to perform a respiratory manoeuvre and at high frequency (1 Hz). The correlation and agreement between β, calculated with and without the use of a respiratory manoeuvre, was then assessed using linear regression analysis and the Bland–Altman technique.

In the PLR subgroup, ventilation rate and V_TI_ were not altered during the study period. The mean values of V_TI_ (ml kg^−1^) and SPeR (%) for 10 consecutive spectral analyses, obtained in three stages of the manoeuvre (‘Legs Down’; ‘Legs Up’; ‘Legs Down’) were used to calculate the slope of β in the manner described above. The initial ‘Legs Down’ data was that obtained in the 10 s immediately before leg elevation. The ‘Legs Up’ data was that obtained in the 10 s commencing at least 35 s after leg elevation. The final ‘Legs Down’ data was that obtained in the 10 s commencing at least 35 s after leg lowering.

Simple linear regression was used to examine the relationship between β and the MAP.

A value of p < 0.05 was considered statistically significant for all comparisons and, where appropriate, 95% confidence intervals and/or mean values ± standard deviations were calculated.

## Results

In all 30 patients who underwent AVR, there was a linear relationship between SPeR and V_TI_:

$${\text{SPeR}}={{\upbeta}}*{{\text{V}}_{{\text{TI}}}}$$

**Fig. 2:**
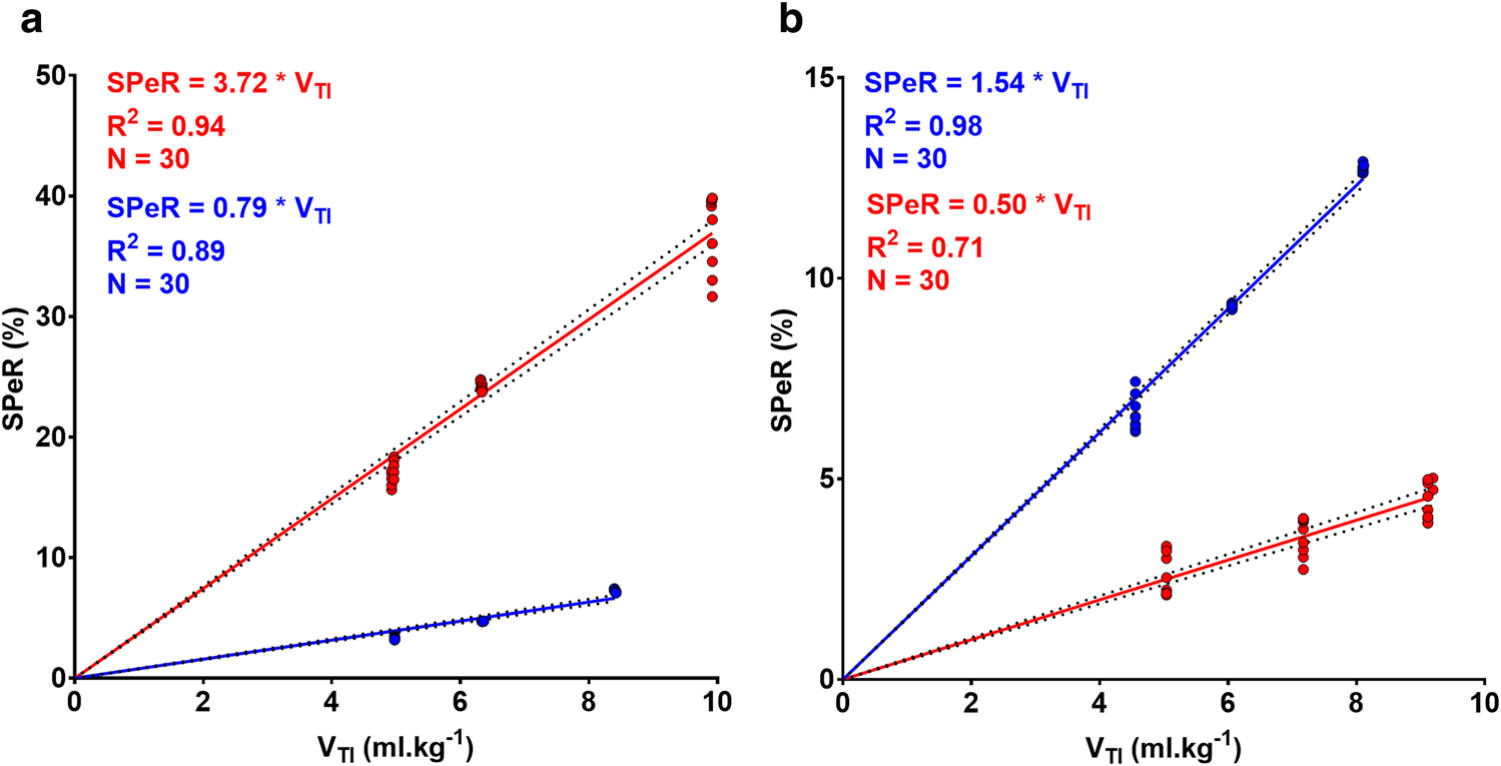
**a** Regression analysis of SPeR and V_TI_ in a patient with severe aortic regurgitation (VCW = 6.6 mm). The regression slope obtained before valve replacement (blue) increased significantly after replacement (red) (p < 0.0001). **b** Regression analysis of SPeR and V_TI_ in a patient with severe aortic stenosis, but no aortic regurgitation (VCW = 0 mm). The regression slope obtained before valve replacement (blue) declined significantly after replacement (red) (p < 0.0001). Note the different scales for SPeR in graphs **a** and **b**

The value of β varied between 0.46 and 4.33 (1.68 ± 0.79). In 12 of the 30 patients, β decreased significantly after AVR and in the remainder it increased significantly. In all cases the change in β was highly significant (p < 0.0005). Typical examples of these changes are shown in Fig. 2.

**Fig. 3:**
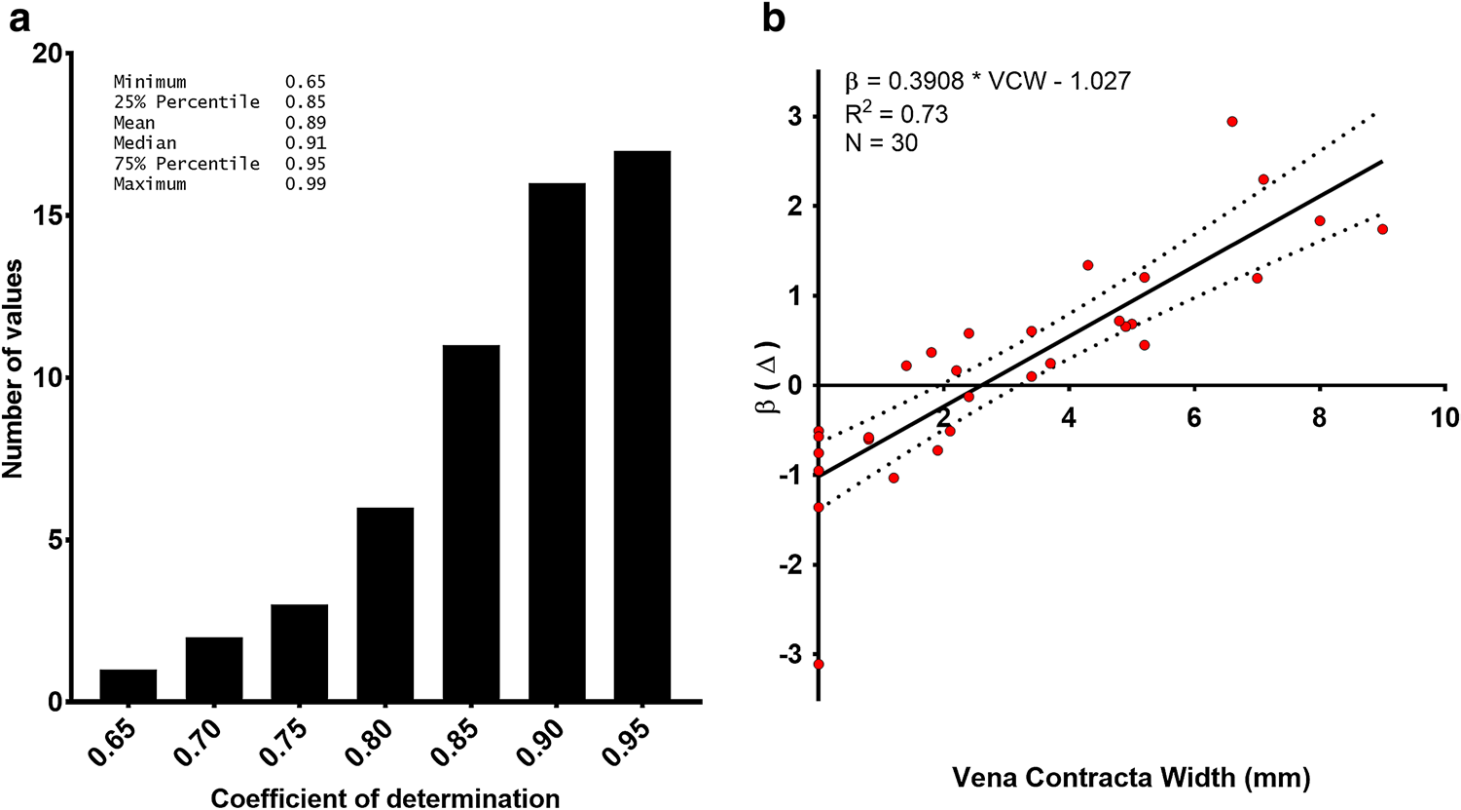
**a** Frequency histogram of the coefficients of determination of the 60 regression analyses, obtained using a respiratory manoeuvre, before and after AVR. **b** Relationship between the pre-replacement VCW and the change in slope of the regression line (β) before and after AVR. In patients with significant aortic regurgitation (VCW > 3 mm), the regression slope consistently increased after AVR, whereas in those with ‘pure’ stenosis, the slope consistently decreased

The coefficient of determination for each of the 60 regression lines which were obtained varied between 0.65 and 0.99 (0.89 ± 0.8). The frequency histogram for these 60 coefficients is shown in Fig. 3a.

The mean value of β before AVR was 1.58 ± 0.78 and after AVR 1.79 ± 0.8. This difference was not significant (p = 0.39). However, when the effect of preoperative aortic regurgitation was examined, a strong correlation between the VCW and the magnitude and direction of change in β was found (β = 0.39 * VCW − 1; R^2^ = 0.73) (Fig. 3b).

**Fig. 4:**
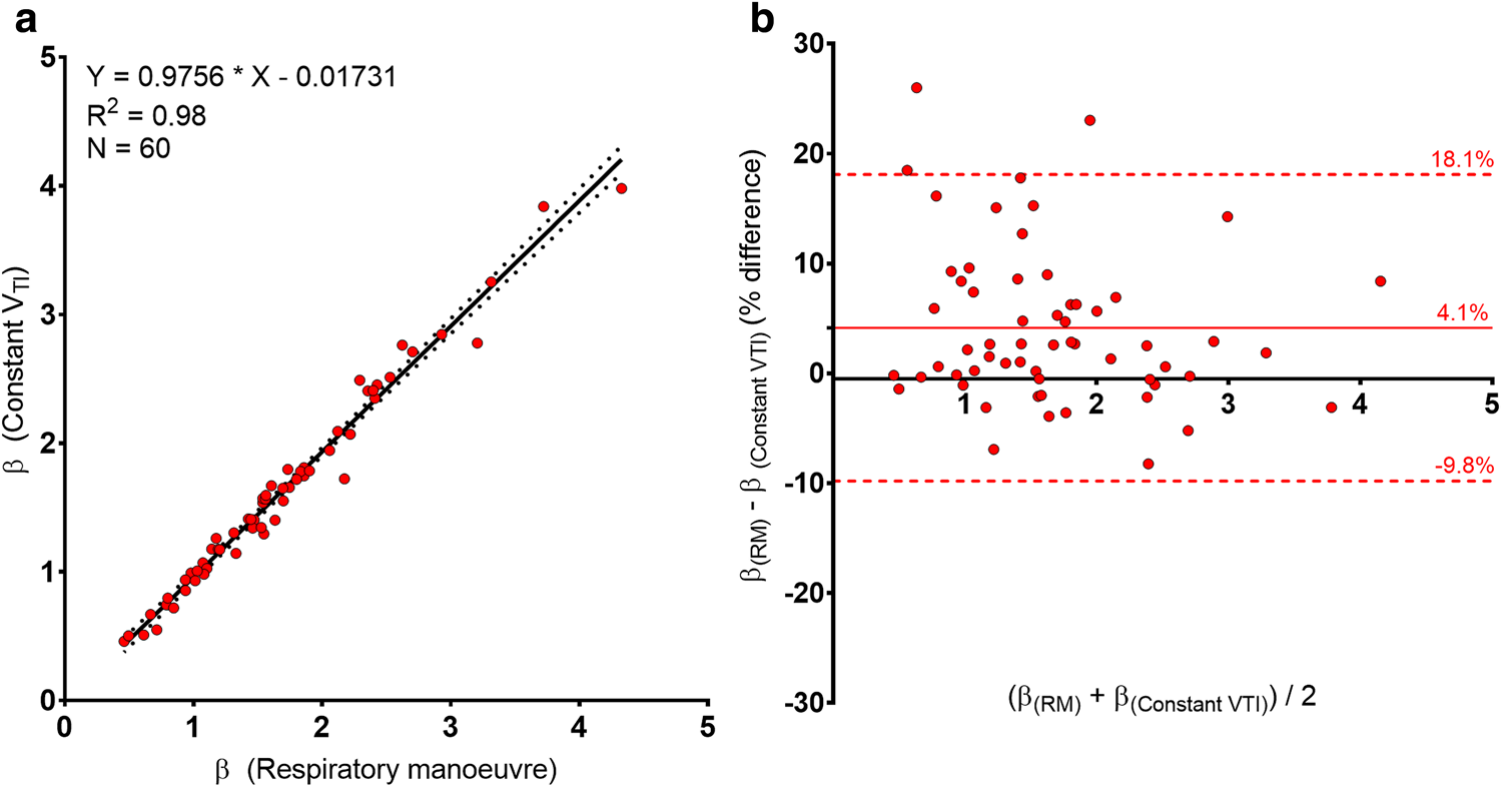
**a** Correlation between the slopes of the SPeR: V_TI_ regression line (β) when obtained using a constant V_TI_ compared with that obtained using a respiratory manoeuvre. **b** Bland–Altman analysis of the agreement between the slopes of the regression line (β) calculated using a respiratory manoeuvre and that obtained at a constant value of V_TI_. The bias (solid red line) and 95% limits of agreement (dotted red line) are shown

The correlation between β determined with and without the use of a respiratory manoeuvre is shown in Fig. 4a (β_ConstVTI_ = 0.98 * β_Resp Man_ − 0.02). The Bland–Altman analysis showed that there was a bias of 4.1% towards the ‘Constant V_TI_’ method of calculation and the 95% limits of agreement were between 18.1 and − 9.8% (Fig. 4b).

For all 60 respiratory manoeuvres which were performed, the average value of low-range V_TI_ was 5.00 ml kg^−1^ ± 0.38 and the average value of mid-range V_TI_ was 7.04 ml kg^−1^ ± 0.69. The corresponding low- and mid-range SPeR values were 7.87 ± 4.1 and 11.53 ± 5.7 respectively. This difference was highly significant (p < 0.0001). In all manoeuvres, the SPeR obtained at the mid-range of V_TI_ exceeded that obtained at the low-range of V_TI_.

**Fig. 5:**
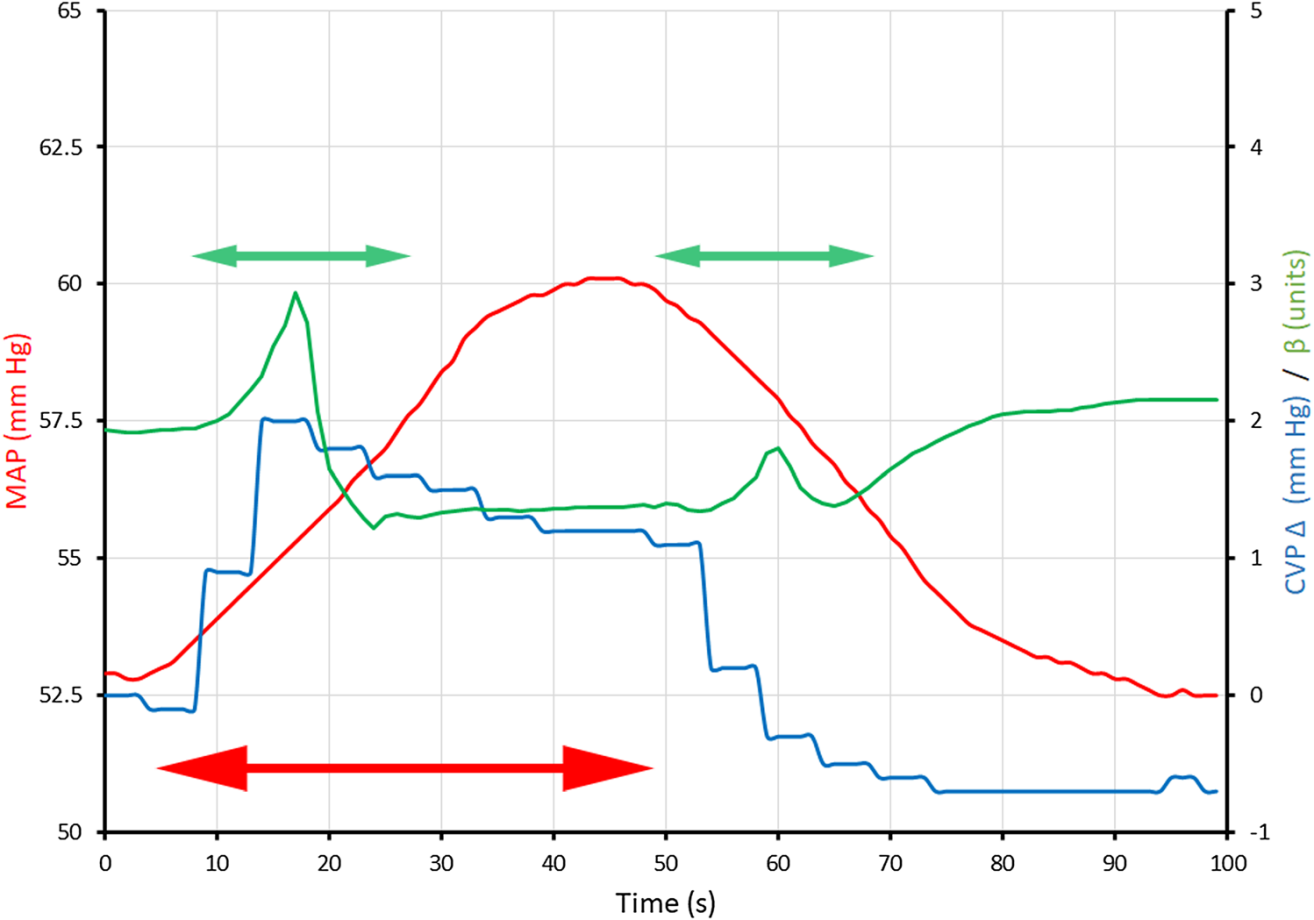
The changes in β (solid green line), CVP (solid blue line) and MAP (solid red line) during PLR in one patient. The solid red arrow corresponds to the period of leg raising. During this time, MAP rises from about 52 to 60 mm Hg; CVP increases sharply by about 2 mm Hg before declining over the ensuing 30 s by about 1 mm Hg; β falls from about 2.0 to 1.3. The changes are reversed on leg lowering. The two green arrows represent periods of spectral analysis during which low frequency artefact (induced by the rapidly changing blood pressure) is apparent. These artefacts were found in all patients and lasted for approximately 20 s

In the PLR subgroup the average V_TI_ during leg-raising was 6.68 ± 0.54 ml kg^−1^. The response of a typical patient to the manoeuvre is shown in Fig. 5.

**Fig. 6:**
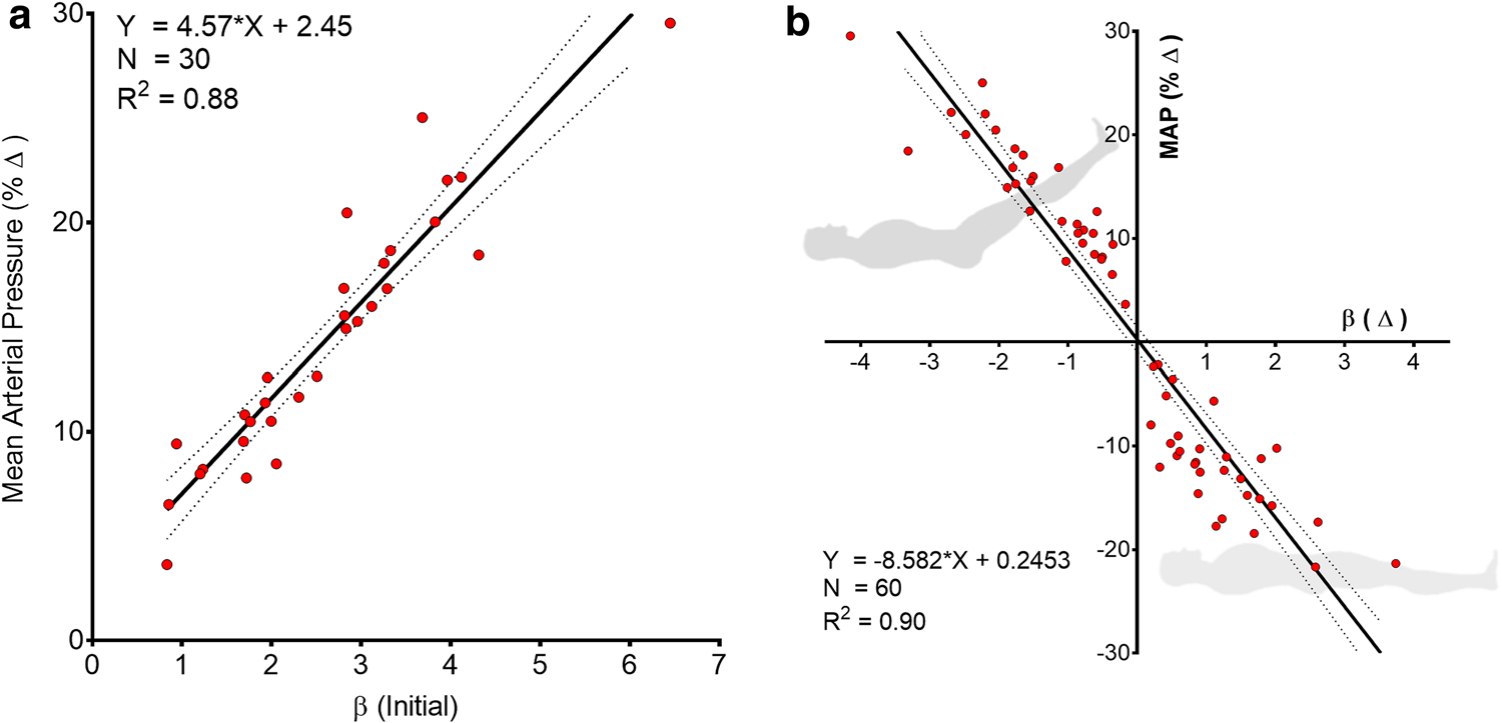
**a** Relationship between the initial regression line slope (β) in the ‘Legs Down’ position and the increase in MAP during subsequent leg raising. **b** Relationship between the change in regression line slope [β (Δ)] and the change in MAP [MAP (%Δ)] following leg raising and lowering. When the legs are raised, β falls and MAP rises, conversely, when the legs are lowered, β rises and MAP falls

In all 30 patients MAP rose and β decreased during leg-raising. There was a strong correlation between the *initial* value of β and the *change* (Δ) in MAP which followed leg-raising (ΔMAP = 4.57 * β + 2.45; R^2^ = 0.88) (Fig. 6a) and a similarly strong relationship between the *change* in β and *change* in MAP which followed either leg-raising or leg-lowering (ΔMAP = − 8.58 * Δβ + 0.25; R^2^ = 0.90) (Fig. 6b).

**Fig. 7:**
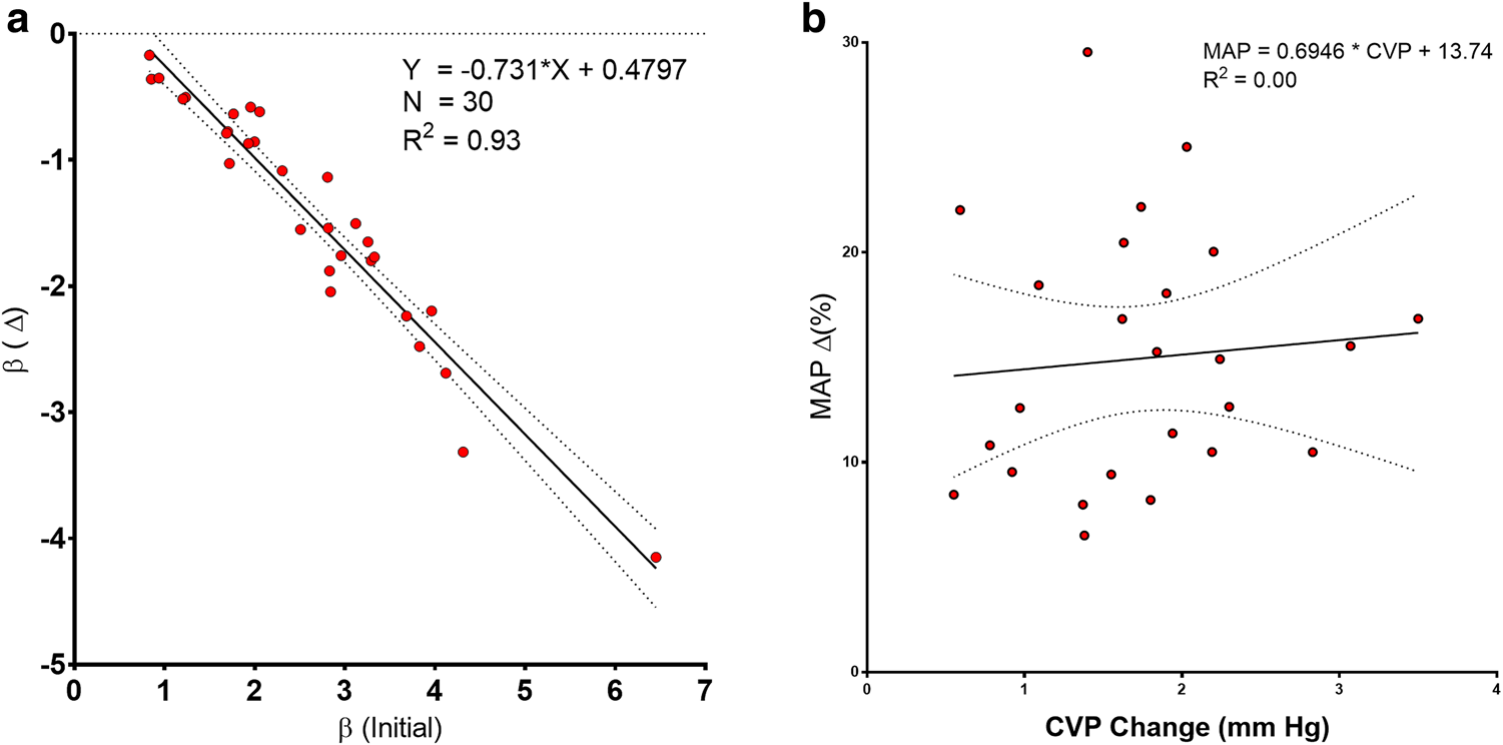
**a** Relationship between initial regression line slope (β) and the change in β following leg raising. There was a strong correlation between the initial value of β and the fall in β which occurred during leg-raising. **b** Lack of relationship between the change in CVP induced by leg raising and the change in MAP

There was also a strong inverse correlation between the *initial* value of β and the *change* in β which accompanied leg-raising (β = − 0.73 * Δβ + 0.48; R^2^ = 0.93) (Fig. 7a).

However, there was no correlation between the *change* in CVP and the *change* (Δ) in MAP which accompanied leg-raising (MAP = 0.69 * CVP + 13.74; R^2^ = 0.00) (Fig. 7b).

## Discussion

### General observations

Three general findings emerged from this study:

First, the feasibility of real-time, spectral analysis of the arterial pressure waveform using a mobile computer was established. FFT of the arterial waveform are usually performed ‘offline’ on a signal acquired by a dedicated, high-frequency (500–2000 Hz), analog-to-digital converter and high spectral resolution is achieved by using a sample window of at least 60 s [9, 10]. However, given that there is a roughly tenfold difference between the respiratory (~ 0.15 Hz) and cardiac (~ 1.5 Hz) peak frequencies, this degree of spectral resolution may be unnecessary for the quantification of their relative peak sizes.

This is borne out in the present study which demonstrates the efficacy of the analysis of a low-frequency (100 Hz) pressure signal using a sample window size of only 30 s. The frequency was chosen because the ‘Aisys’ monitor streams the digitised signal in arrays which correspond to this sample rate and the window size and windowing technique were determined empirically in the pre-clinical testing phase. (The demonstration program allows the user to examine the effects of varying sample window size and/or windowing technique using either their own data or the raw data files provided.) Alian’s finding [16] that spectral analysis could be performed successfully on an arterial pressure waveform sampled at 100 Hz and Thiele’s method for the calculation of SPeR [9] were both confirmed. However, the SPeR obtained in the present study differed slightly from that calculated by Thiele because of the different signal processing techniques used during spectral decomposition.

The second general finding was that, during IPPV, there was a linear relationship between SPeR and V_TI_ and that the slope of this relationship (β) changed significantly and consistently in response to changing haemodynamic conditions. The influence of changes in V_TI_ on PPV (and stroke volume variation) has been demonstrated in several time-domain-based studies [5–8]. However, the lack of precision of time-domain-based measurements—particularly at tidal volumes of less than 8–10 ml kg^−1^—does not allow for a detailed examination of the effect [6] and neither the linearity of the relationship nor the use of a change in the slope of the relationship as a measure of the cardiac response to changing ventricular inflow has been clearly demonstrated using time-domain-based techniques. In spectral (frequency-domain) analysis, the requirement to perform analyses on a consecutive sequence of breaths provides an index which represents the ‘average’ SPeR of all breaths in the sample window. As a result, it becomes possible to measure the effect of tidal ventilation on the SPeR more accurately and the precision of the measurement is such that the impact of ventilation at ~ 5 ml kg^−1^ and tidal volume changes of ~ 2 ml kg^−1^ can be detected quite easily.

The demonstration that β could be determined at a constant level of V_TI_ is important because it allows the calculation to be made repetitively, at high frequency, without the requirement for any change in tidal ventilation. Thus, in the PLR subgroup, where haemodynamic change occurs quite rapidly, it was possible to follow the changes in β at 1 Hz (Fig. 5).

The third general finding was that significant changes in SPeR were demonstrable at low tidal volumes (~ 5 ml kg^−1^). In the AVR subgroup, significant differences in SPeR were consistently demonstrated when tidal volume was increased from 5 to 7 ml kg^−1^ and in the PLR subgroup, significant differences in SPeR, induced by leg raising, were consistently found using tidal volumes of about 6.7 ml kg^−1^ (6.68 ± 0.54 ml kg^−1^). This sensitivity of SPeR to changing tidal volume at low tidal volume is also well-shown in Video 1, where a patient has been ventilated at five different values of V_TI_ in the range 5–8 ml kg^−1^ and yet the different peak ratios at each level of tidal volume remain quite distinct. The sensitivity and precision of the technique was also attested to by the very high values of the coefficient of determination for the SPeR: V_TI_ relationship. In the 60 datasets reported here, the coefficient was more than 0.80 in 85% of cases and more than 0.90 in over 50% of cases.

**Fig. 8:**
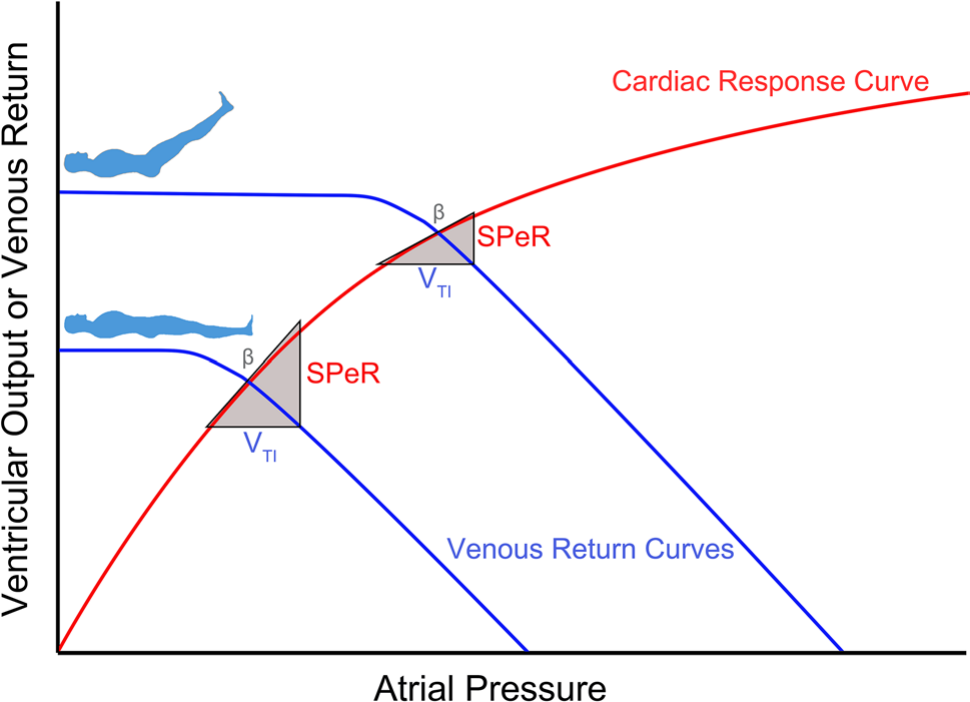
Guyton diagram at two hypothetical levels of venous return (blue). As venous return increases, the equilibrium point with the cardiac response curve (red) is shifted upwards and to the right and SPeR and β at the new equilibrium point decline

The physiological mechanism which underlies PPV in the time domain and the SPeR: V_TI_ relationship in the frequency domain is the cyclical change in atrio-ventricular inflow which accompanies tidal ventilation. During IPPV, it has been shown that cyclical variations in left atrial area of about ± 12% and left ventricular end-diastolic volume of about ± 8% can be found in patients ventilated at 7–9 ml kg^−1^ [17]. So it is that IPPV provides pattern of oscillatory atrio-ventricular inflow which is well-suited to spectral analysis and which provides a convenient method for exploring the interaction between the venous return and the cardiac response curves of the ‘traditional’ Guyton model [12]. In this context, V_TI_ can be regarded as a surrogate for changing *venous return*, SPeR as a surrogate for changing *stroke volume*, and β, the slope of the SPeR: V_TI_ relationship, as a surrogate for the *slope of the cardiac response curve* at its equilibrium point with the venous return curve (Fig. 8).

There are various time-domain metrics which bear some equivalence to β in this study. In particular ‘dynamic’ arterial elastance (E_ADyn_) has been used to describe the dynamic response of the arterial system to changes in stroke volume [18, 19]. However, β has a wider scope than E_ADyn_ in that it also reflects the ability of the ventricle to translate an increase in venous return to an increase in stroke volume.

Transient increases in venous return to the left ventricle are accommodated by increases in left atrial pressure and/or stroke volume and, the extent to which pressure or volume changes predominate, is determined by arterial elastance and impedance and ventricular systolic and diastolic function at the time of the change in venous return.

In the hypothetical example shown in Fig. 8, two levels of venous return found during a PLR manoeuvre at constant V_TI_ are illustrated.

In the supine position, the intersection of the venous return curve and cardiac response curve is shown at a point where the ventricular–arterial system can accommodate the cyclical increases in left heart inflow induced by tidal ventilation with relatively little increase in left atrial pressure. As a result, both SPeR and β are relatively high and the cyclical increase in atrial pressure is relatively low.

In contrast, during leg-raising, the equilibrium point of the venous return curve on the cardiac response curve has moved upwards and to the right. The ventricular–arterial system is now less able to respond with increased forward flow when the same cyclical increase in left heart inflow is applied. As a result, a greater proportion of the increased venous return is retained in the atrium and pulmonary venous system, these capacitances are more highly charged, and the increase in atrial pressure is relatively greater. In consequence, SPeR and β are now lower than in the supine position. Clearly, this ability to translate changes in venous return to changes in stroke volume varies both within patients and between patients (as haemodynamic conditions change).

At its simplest level, it is manifest in the finding that patients can be classified as ‘responders’ or ‘non-responders’ to fluid loading [3]. However, the determinants of responsiveness are complex and, as stated above, are ultimately determined by the interaction between the arterial and atrio-ventricular systems.

### Specific findings in the AVR subgroup

Several groups have described the changes in E_A_ which occur after AVR. If it is accepted that arterial elastance is a parameter of the SPeR, and that VCW is a good (numerical) metric for the assessment of aortic regurgitation [13], then the findings in the AVR subgroup are consistent with the changes found by others.

In a simulation study, data from 45 patients with AR was used to supply the parameters of a sophisticated computer model of ventricular–arterial interaction. The authors were able to demonstrate that the severity of AR was the main determinant of E_A_ and that repairing the aortic valve resulted in a significant increase in post-repair elastance [20].

In an intraoperative study of 9 patients with AR, using a technique which combined intraventricular catheterisation with echocardiography, E_A_ was found to increase significantly after valve replacement (p = 0.01) [21].

Finally, in an echocardiographic study of over 200 patients who underwent AVR, E_A_ increased significantly after AVR in those with AR (p < 0.01), was unchanged on those with mixed aortic disease, and decreased in those with isolated aortic stenosis (p < 0.01) [22].

These observations are consistent with the finding that the direction and magnitude of change in β after valve replacement is strongly correlated with the preoperative VCW and suggest that the change in β reflects the change in arterial elastance which accompanies AVR.

### Specific findings in the PLR subgroup

The concept of ‘Functional Haemodynamic Monitoring’ has been described as “The process of assessing the dynamic response of a measured hemodynamic variable to a defined, reproducible, and readily reversible extrinsic stress” [23]. PLR is widely regarded as a reliable method for the induction of a transient increase in ventricular pre-load [24–28] and it provides a good example of such a reversible extrinsic stress (Fig. 5**)**.

Three significant findings emerged from this subgroup:

First, β was consistently significantly higher in the legs-lowered position than the legs-raised position (Figs. 5, 6b).

Second, the greater the initial value of β (before leg-raising), the greater the increase in MAP which occurred on leg-raising (Fig. 6a) and the greater the decline in β that accompanied this increase in pressure (Fig. 7a).

Finally, in all cases, significant results were obtained at a V_TI_ of less than 8 ml kg^−1^ (6.68 ± 0.54 ml kg^−1^) rather than the value of greater than 8 ml kg^−1^ which is generally recommended for use with the time-domain-based techniques [5].

In two time-domain-based studies [24, 25] on the effect of PLR on PPV in mechanically ventilated patients, PPV fell significantly during leg-raising in all 42 patients studied. In several other time-domain-based studies on ventilated patients, the direct effect of volume expansion on PPV has been measured [29–31]. In all these studies—which involved over 100 patients, PPV fell significantly after volume expansion.

The finding that β, in the present study, fell significantly in the legs-raised position is consistent with these observations and is also consistent with the view that the increased venous return induced by leg-raising moves the venous return equilibrium point on the cardiac response curve upwards to a position where the slope of the response curve is less (Fig. 8).

The PLR study also incidentally demonstrated the ease with which other monitored variables (such as CVP) can be integrated synchronously with the calculated spectral data (Fig. 5).

### Technique merits and limitations

The technique has both computational and practical advantages over other methods of PPV analysis. Computationally: the calculations do not require the use of specialised hardware or a powerful CPU; Fourier transformations can be implemented using only a few lines of C# code; and β can be calculated at a high frequency and is easily integrated into the monitor’s data stream.

The main practical advantage is that, unlike the time-domain-based analyses of PPV, the use of high tidal volume ventilation is not essential [6].

However, the technique suffers from two significant limitations:

First, it can only be used in patients who are in regular sinus rhythm. If a single ectopic beat occurs during the sampling period, it will markedly distort the spectral analysis and the disruptive effect will be apparent for the duration of the sample window. Doubling the sample window length will not negate this effect. The technique is unusable in patients in atrial fibrillation at any sample window length.

Second, the measurements are most reproducible when performed in ventilated patients undergoing constant rate, VCV. Constant rate, pressure-controlled ventilation can also yield acceptable results provided that tidal volume does not change markedly during the analytical period. During spontaneous ventilation, respiratory and cardiac peaks can be easily detected, however the results are of little use unless the patient is breathing regularly at a more-or-less constant tidal volume.

It should also be noted that, in this study, no attempt was made to compare SPeR with ‘PPV’ as measured by the monitor. The ‘Aisys’ monitor calculates ‘PPV’ simply as the percentage change in *systolic* pressure, over an unspecified time, rounded to the nearest whole number [32] and does not implement a complex, validated, algorithm such as that described by Aboy et al [33], which is available on other monitoring systems (‘Intellivue MP70’, Philips Medical Systems, Böblingen, Germany).

### Conclusion

Preliminary experience with the technology described here suggests that real-time spectral analysis of physiological waveforms, using a ‘smart’ device connected wirelessly to a standard monitoring system, merits further evaluation.

Clearly, the results of this pilot study require confirmation and the hypothesis that β represents the slope of the cardiac response curve at its equilibrium point on the venous return curve needs further testing. Comparisons between frequency- and time-domain analyses of PPV using techniques which include the formal measurement of stroke volume are also required.

Although there are valid concerns regarding the use of clinical monitors as scientific instruments [34], it has recently been proposed that anesthesiologists become more effective ‘frontline physiologists’ by developing new methods of clinical waveform analysis, using real-time data acquired from such monitors [16]. The techniques described here enable such forms of analysis and may facilitate our transition to the ‘frontline’ of physiological research.

## Electronic supplementary material

Below is the link to the electronic supplementary material.

Supplementary material 1 (XML 448 KB)

Supplementary material 2 (XML 339 KB)

**Video 1** Screen capture of data acquisition and analysis during a respiratory manoeuvre (MP4 4924 KB)

Supplementary material 4 (MSI 6688 KB)

Supplementary material 5 (PDF 131 KB)
