# Supplementary material for: Real-time, spectral analysis of the arterial pressure waveform using a wirelessly-connected, tablet computer: a pilot study
Source: J Clin Monit Comput. 2018 Apr 28;33(1):53–63. doi: 10.1007/s10877-018-0145-0 (PMC6314984; doi:10.1007/s10877-018-0145-0)
Supplement: Supplementary file 5 — Supplementary material 5 (PDF 131 KB) [file 10877_2018_145_MOESM5_ESM.pdf]

Supplementary Digital Content:

EQUIPMENT:	
**Transducer system:**<br>IcuMedical<br>951 Calle Amanecer,<br>San Clemente,<br>CA 92673 USA<br>Reference Number: 011-46104-33	**Arterial Cannula:**<br>Arrow International,<br>2400 Bernville Road,<br>Reading,<br>PA 19605 USA<br>Reference Number: RA-04220
**RS232 serial adapter:**<br>Grid Connect, Inc.<br>1630 W. Diehl Road<br>Naperville, Illinois 60563 USA<br>Part Number: GC-BT-FIREFLY-DTE-NOPS	

Programming environment:

The following computing packages were used in the development of the applications:

- Microsoft Visual Studio 15. Release 15.2.0+26430.13
- Microsoft .NET Framework Version 4.7.02556
- Xamarin 4.5.0.486 (fec6f88)
- MathNet.numerics 4.0.0 (beta 07)

The author wrote his own interface to the anaesthesia monitor. However, an open source version can be found at:

- https://sourceforge.net/projects/vscapture/

See: Karippacheril JG, Ho TY. Data acquisition from S/5 GE Datex anesthesia monitor using VSCapture: An open source.NET/Mono tool. J Anaesthesiol Clin Pharmacol. 2013 Jul;29(3):423-4.

Useful Websites:

Website:	URL:	Last Accessed:
Microsoft 'Visual Studio'	https://www.visualstudio.com/	5 February 2018
Xamarin 'Studio'	https://xamarin.com/	5 February 2018
MathNet 'Numerics'	http://numerics.mathdotnet.com/	5 February 2018
VS 'Capture'	https://sourceforge.net/projects/vscapture	5 February 2018
Graphpad 'Prism'	https://www.graphpad.com/demos	24 February 2018

Installing the 'Prism' viewer:

A fully-functional version of Graphpad 'Prism' is available at the website listed above. 30 days after installation it reverts to 'Viewer' mode (unless a subscription is purchased).

Sine wave generator:

The application was initially developed using a series of data files which were created by a purpose-written, composite sine wave generator written in C# and running as a 'Windows Forms' application.

The generator created a simulated arterial trace (with respiratory variability) by summing three sine waves to which was then added a DC bias.

The application initially created a 'primary' (Pulse) wave with an adjustable frequency in the range 1 to 3 Hz and an adjustable amplitude.

In order to simulate 'sinus arrhythmia' or some other cause of an pulse irregularity, the frequency of this primary wave could also be set to be randomly varied by up to +/- 5% at the time of the generation of each wave cycle.

In order to simulate the dicrotic notch, an amplitude-adjustable 'secondary' wave (which was the second harmonic of the primary wave frequency) was added to the primary wave.

Systolic pressure variability was simulated by the addition of a frequency- and amplitude- adjustable tertiary wave. The frequency range of this wave was 0.12 – 0.2 Hz.

Finally, the effect of changing systemic vascular resistance was simulated by the addition of a variable DC bias to the summed waves.

The generator wrote the data to a file as a sequence of ‘Datex-Ohmeda Record’ structures.

During development, the application then read these data as ‘Datex Ohmeda Records’ at a frequency of 1 Hz.

Fourier analysis:

Interested readers will find a facsimile of Fourier’s original paper at:

http://gallica.bnf.fr/ark:/12148/bpt6k33707/f220n7.capture

Installing the application:

1. Download the file named AisysFFT.msi
2. Double click it
3. Follow the wizard’s instructions

This ‘Live’ version of the application only works on ‘Windows’ computers which are either hardwired or ‘bluetooth’ connected to GE ‘AISYS’ monitors.

The application is supplied with two sample raw data files which can be used in ‘File’ mode and will also work with other ‘raw’ GE data files.

C# snippet for FFT using MathNet package:

```
void FFTCalculateAsync()
{
  FFTInputArray = DeTrendInputArray(FFTInputArray); //REMOVE DC OFFSET
  Complex[] complexInput = new Complex[FFT_SAMPLE_SIZE];
  double FFTWindow;
    for (int i = 0; i < complexInput.Length; i++)
      {
        FFTWindow = (double)FFTInputArray[i] * FFTWindowFunction[i];
        Complex complexValue = new Complex(FFTWindow, 0);
        complexInput[i] = complexValue;
       }
   MathNet.Numerics.IntegralTransforms.Fourier.Forward(complexInput);
    for (int i = 0; i < complexInput.Length; i++)
      {FFTMagnitudeArray[i] = (int)Complex.Abs(complexInput[i]);}
  }
```
